# Periodontal connection with intestinal inflammation: Microbiological and immunological mechanisms

**DOI:** 10.1111/prd.12424

**Published:** 2022-03-04

**Authors:** Sho Kitamoto, Nobuhiko Kamada

**Affiliations:** ^1^ Division of Gastroenterology and Hepatology Department of Internal Medicine University of Michigan Ann Arbor Michigan USA

**Keywords:** inflammatory bowel disease, oral bacteria, oral‐gut axis, periodontitis, systemic interactions

## Abstract

Humans have coevolved with the trillions of resident microbes that populate every nook and cranny of the body. At each site, the resident microbiota creates a unique ecosystem specialized to its environment, benefiting the development and maintenance of human physiology through harmonious symbiotic relationships with the host. However, when the resident microbiota is perturbed, significant complications may arise with disastrous consequences that affect the local and distant ecosystems. In this context, periodontal disease results in inflammation beyond the oral cavity, such as in the gastrointestinal tract. Accumulating evidence indicates that potentially harmful oral resident bacteria (referred to as pathobionts) and pathogenic immune cells in the oral mucosa can migrate to the lower gastrointestinal tract and contribute to intestinal inflammation. We will review the most recent advances concerning the periodontal connection with intestinal inflammation from microbiological and immunological perspectives. Potential therapeutic approaches that target the connection between the mouth and the gut to treat gastrointestinal diseases, such as inflammatory bowel disease, will be examined. Deciphering the complex interplay between microbes and immunity along the mouth–gut axis will provide a better understanding of the pathogenesis of both oral and gut pathologies and present therapeutic opportunities.

## 1 INTRODUCTION

The surface of our body is covered by numerous commensal microorganisms, including bacteria, fungi, and viruses. The oral cavity has the second largest commensal bacterial community, harboring over 770 species of bacteria that live in different habitats, including the lips, teeth, tongue, cheeks, and palate.^1^ Oral bacteria are primarily members of the phyla Firmicutes, Fusobacteria, Proteobacteria, and Actinobacteria, creating complex ecosystems by adapting to each unique environment.^2^ Although the role of the commensal oral bacteria in oral health is yet to be fully understood, the colonization of the bacteria in the oral cavity after birth appears to be essential for the development of the oral mucosal immune system and terminal maturation of the stratified oral epithelium, which is crucial to the establishment of oral mucosal homeostasis.^3^ Also, certain types of commensal oral bacteria serve as the first‐line of defense against the colonization of exogenous pathogens by inhibiting the adhesion of pathogens and the production of bactericidal products (eg, bacteriocins, hydrogen peroxide).^4^

Like the oral compartment, unique environments in the human gut (eg, nutrient and anaerobic conditions) shape a complex gut microbiota, consisting of the collection of trillions of microbial cells with thousands of bacterial species. It is the largest bacterial community in the human body and plays an essential role in host physiological homeostasis, including the education of the host immune system, nutrient digestion, and defense against colonization by pathogenic microorganisms.^5^, ^6^, ^7^, ^8^ Because of its fundamental role in controlling intestinal physiology, disturbance of the gut microbiota, often referred to as gut dysbiosis, has been demonstrated to underlie multiple intestinal pathologies, including irritable bowel syndrome, inflammatory bowel disease (IBD), and colorectal cancer (CRC). The advances in sequencing technologies have revealed an abnormal enrichment of typical oral resident bacteria in the luminal contents and the mucosal tissues of the gut in patients with gut pathologies.^9^ Given the studies depicting the pathological impact of certain oral resident bacteria (eg, *Porphyromonas gingivalis* and *Fusobacterium nucleatum*) on gut homeostasis, it is conceivable that the oral cavity serves as a reservoir of oral pathobionts whose ectopic gut colonization contributes to the intestinal pathologies. Studies have clearly shown that patients with gut inflammation, such as IBD, exhibit a significant enrichment of oral bacteria in the gut, including pathogens associated with the oral inflammatory disease periodontitis.^10^, ^11^ This notion is supported by studies showing the distinct oral microbiota^12^ and increased prevalence of periodontitis in IBD patients when compared with healthy individuals.^13^ These observations may be indicative of the link between periodontal and gut inflammation established through microbial communications.

## 2 POTENTIAL ROUTES OF GUT TRANSLOCATION OF ORAL BACTERIA

The translocation of oral bacteria from the oral cavity to the gut mucosa is poorly defined. Two potential routes have been proposed.

### 2.1 Hematogenous dissemination

Oral resident bacteria can disseminate systemically by the hematogenous route originating in the oral cavity. In this regard, mechanical injuries in the oral cavity can lead to the spread of oral bacteria into the systemic circulation.^14^, ^15^ Moreover, oral bacteria such as *P gingivalis* are found in the blood collected from patients with periodontal diseases, including periodontitis.^16^ Consistently, ligature‐induced murine periodontitis leads to oral bacterial dissemination to the liver and spleen, indicating that the hematogenous spread of oral bacteria can be determined by oral disease status.^17^ Furthermore, it has been shown that hematogenously inoculated *Fusobacteria* strains are more successful in tumor colonization in the gut than gavaged strains, suggesting the importance of the circulatory system as a route of oral bacteria dissemination.^18^ Oral bacteria are also known to invade and survive inside dendritic cells and macrophages, implying the hijacking of host immune cells to serve as Trojan horses for the dissemination of bacteria from the oral to the gut compartment.^19^

### 2.2 Enteral dissemination

People swallow about 600 times a day and produce ~1.5 L of saliva containing 1.5 × 10^12^ oral bacteria.^20^, ^21^ Although more than half of the oral resident bacterial species (eg, *Streptococcus* spp. *Veillonella* spp.) are detectable in the gut, implying oral–gut translocation of oral bacteria even in healthy individuals,^22^ oral bacteria are generally poor colonizers in a healthy gut. This is due to the segregation of mouth and gut bacterial communities through the multiple barriers conferred by the gastrointestinal tract.^9^ The first barrier against the oral bacterial translocation to the gut is gastric acidity.^23^, ^24^ It is estimated that over 99.9% of swallowed bacteria of oral origin cannot survive in the stomach due to its acidic antimicrobial environment, which reduces bacterial numbers by 5‐6 orders of magnitude.^21^, ^25^ In line with this notion, a significant elevation of gut colonization by oral bacteria (eg, *Streptococcus* spp., *Veillonella* spp., *Haemophilus* spp.) occurs in patients who have gastric achlorhydria caused by the long‐term use of proton pump inhibitors (PPIs). Consistently, patients with gastroesophageal reflux disease treated with long‐term PPI therapy also exhibit a higher oral bacterial accumulation in the gut compared to healthy individuals.^26^ Further, individuals who have gastritis after gastric surgery (eg, gastric bypass or removal) exhibit an altered gut microbial composition, accompanied by the accumulation of resident oral bacteria in the gut (eg, *Streptococcus* spp., *Veillonella* spp., and *Enterobacteriaceae*).^27^, ^28^ Of note, the attenuated gastric acidity is observed in patients with IBD, indicating the potential contribution of a “leaky stomach” in facilitating a profound colonization of oral bacteria in the gut.^29^ Importantly, certain types of oral pathogens, such as *P gingivalis*, can tolerate the acidic environment in the stomach and pass through the stomach barrier.^30^ Consequently, although possibly less effective for those bacteria that can tolerate the acidic environment, the prevention of the enteral transmission of oral bacteria by gastric acids is considered as the primary defense mechanism. Secondary, given the colonization resistance conferred by the gut resident microbiota,^31^ preservation of the harmonious microbial structure in the gut is also important for preventing ectopic colonization by ingested oral bacteria. This notion is supported by the intestinal expansion of oral bacteria in patients who take certain types of antibiotics (eg, vancomycin), as the antibiotic treatment provokes gut dysbiosis, which generates the niche for ingested oral bacteria.^9^ In addition to antibiotics, multiple factors that cause gut dysbiosis, such as gut inflammation, diets, artificial sweeteners, may also contribute to the opportunistic gut colonization by oral bacteria.^9^

## 3 MICROBIAL PATHWAY (VIA DIRECT GUT COLONIZATION OF ORAL PATHOBIONTS)

**FIGURE 1:**
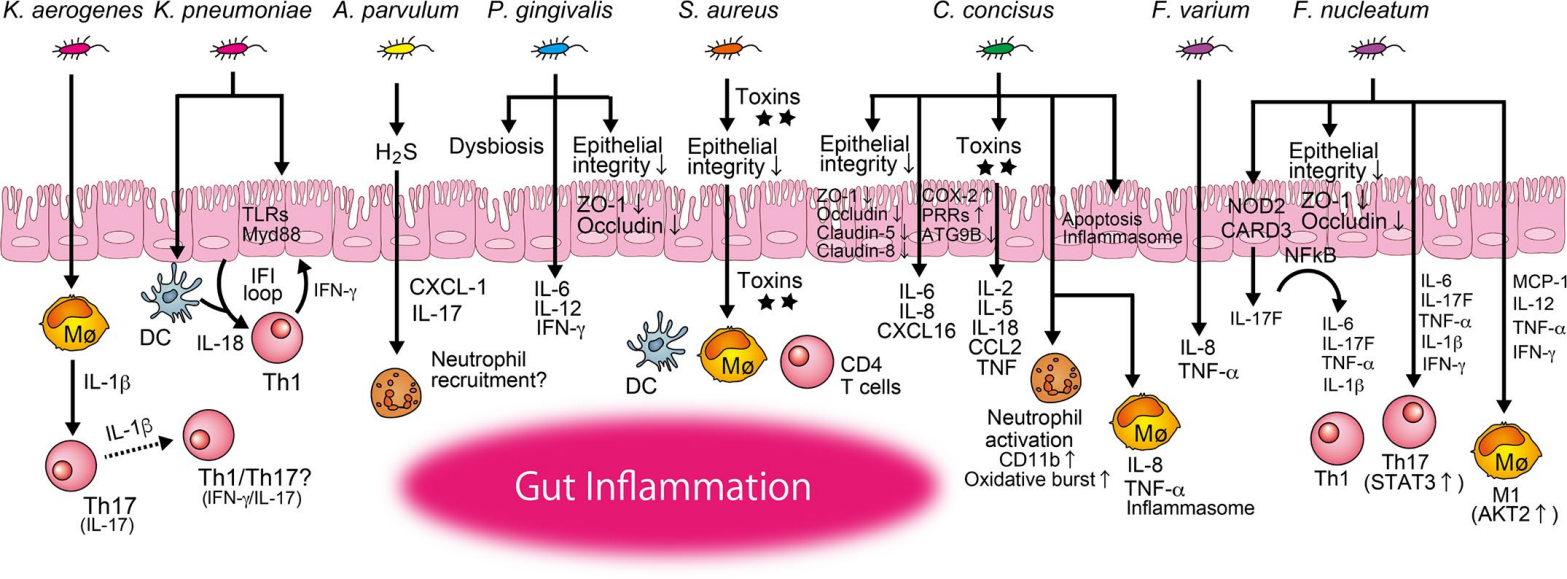
Possible mechanisms of gut inflammation caused by direct colonization by oral pathobionts (microbial pathway). Once oral pathobionts reach the intestine, they first cross the intestinal epithelium. Certain oral pathobionts can adhere to and invade the epithelial cells. The host responses are variable, such as cytoskeletal rearrangement, expression of pattern recognition receptors such as toll‐like receptors (TLRs), inflammasome assembly, cell death, and the release of proinflammatory cytokines. Some oral pathobionts produce cytotoxic substances (eg, hydrogen sulfide [H_2_S], toxins), leading to disruption of the intestinal integrity. A compromised intestinal epithelium allows oral pathobionts, as well as other commensal microorganisms and their metabolites, to move from the lumen to the lamina propria. Oral pathobionts interact with immune cells including macrophages (Mø), DCs, neutrophils, and T cells in the lamina propria, thereby instigating the development of gut inflammation through the activation of multiple inflammatory cascades, including the induction of proinflammatory cytokines and chemokines and the development of pathogenic T cells

Disordered gut microbial distribution and discordant immune responses underlie the development of gut inflammation. Once oral pathobionts colonize the gut, they may be the causative agents, responsible for inducing abnormal immune responses in the gut, thereby leading to intestinal inflammation (Figure 1). Multiple oral resident bacteria are reported to be potential oral pathobionts that are conducive to gut inflammation.

### 3.1 *Fusobacteria* spp.

Certain members of the family *Fusobacteriaceae*, such as *F varium* and *F nucleatum*, are enriched in the gut of patients suffering from IBD, and their abundance is significantly elevated when the disease is active, rather than in remission.^9^, ^32^, ^33^ As genetically identical strains of *F nucleatum* are detectable in both the saliva and colonic tumors of patients with CRC,^34^ *Fusobacterium* strains found in the gut of the IBD patient likely originate from the oral cavity. In addition, considering the inflammatory capacity of *Fusobacteria* spp. in the oral cavity,^35^, ^36^, ^37^ the involvement of oral‐derived *Fusobacteria* spp. in the exacerbation of gut inflammation is plausible. *F varium* can invade the intestinal epithelium and evoke the production of proinflammatory cytokines, such as interleukin (IL)‐8 and TNF‐α, from the intestinal epithelial cells.^38^ Similarly, *F nucleatum* is also highly invasive to intestinal epithelial cells and induces TNF‐α and IL‐1β expression.^39^ Moreover, *F nucleatum* facilitates dextran sulfate sodium (DSS)–induced colitis by disrupting the integrity of the epithelial barrier; reducing tight junction proteins such as ZO‐1 and occludin.^32^, ^33^, ^40^ Activation of the caspase activation and recruitment domain 3 (CARD3)/IL‐17F/nuclear factor‐kappa B (NF‐κB) cascade in the epithelial cells on the colonization of *F nucleatum* also fuels intestinal inflammation through the secretion of proinflammatory cytokines, such as IL‐6, IL‐17F, IL‐1β, and TNF‐α.^32^, ^41^ Further, *F nucleatum* aggravates the progression of DSS‐induced colitis by promoting M1 macrophage polarization through the activation of the AKT2 pathway.^40^ *F nucleatum* also promotes the secretion of proinflammatory cytokines (TNF‐α, IFN‐γ, IL‐1β, IL‐6, and IL‐17) and activates the signal transducer and activator of transcription 3 (STAT3) signaling pathway, thereby inducing the expansion of Th1 and Th17 cells in the DSS‐induced colitis model.^33^ However, the administration of *F nucleatum* to colitis‐associated mouse models (eg, BALB/c IL‐10^−/−^ and BALB/c T‐bet^−/−^ × Rag2^−/−^) neither accelerates gut inflammation nor increases the number of colorectal adenomas.^42^ Although *F nucleatum* is a well‐recognized oral resident bacterium abundant in colonic tumors, and a known contributor to tumorigenesis,^18^, ^43^ its role and the mechanisms involved in the development of gut inflammation remain open to debate.

### 3.2 *Porphyromonas* *gingivalis*

*P gingivalis* is a major periodontopathic bacterium with a wide variety of proinflammatory capacities in the pathogenesis of periodontal diseases, such as periodontitis.^44^, ^45^ Multiple studies have revealed that the orogastric administration of *P gingivalis* to mice may impair epithelial integrity in the gut. For instance, continuous administration of *P gingivalis* (ie, twice a week, for 5 weeks) to C57BL/6N mice causes endotoxemia, accompanied by the decrease of gene expression of tight junction protein ZO‐1 and increase of proinflammatory cytokines IL‐6, IL‐12, and IFN‐γ in the gut.^46^ Similarly, administration of a single oral dose of *P gingivalis* to C57BL/6N mice results in the reduced expression of intestinal tight junction proteins ZO‐1 and occludin in the gut, and the systemic dissemination of enterobacteria to the liver, indicating the disruption of the intestinal barrier function.^47^ Interestingly, the gut microbial composition of mice treated with *P gingivalis* was clearly distinct from that of sham‐treated mice, with the expansion of unclassified *Muribaculaceae* and *Prevotella* spp., which are similar to the IgA‐coated colitogenic pathobionts in the gut.^48^ This indicates that *P gingivalis* itself can be colitogenic, yet gut dysbiosis driven by the colonization of *P gingivalis* may also play a role in the induction or exacerbation of colitis. In a clinical setting, patients with IBD are known to have an increased prevalence of periodontitis compared to individuals who do not have IBD.^13^ Given that large quantities of oral bacteria are constantly swallowed and reach the gut, it is plausible that numerous *P gingivalis*, ranging between 10^6^‐10^8^ cells per mL in subgingival and salivary samples (corresponding to 10^9^‐10^11^ copies daily), are swallowed by patients with chronic periodontitis.^49^ Although the precise impact of gut colonization of *P gingivalis* on intestinal inflammation remains unexplored, its proinflammatory potential suggests that it may exacerbate the inflammation. On the other hand, it is also reported that monocolonization of *P gingivalis* in the gut promotes beneficial changes in the gut immune system, including the elevation of genes related to tight junction proteins and the antiinflammatory cytokine IL‐10.^50^ Further studies would clarify the impact of gut colonization of *P gingivalis* on the pathogenesis of intestinal inflammation.

### 3.3 *Atopobium* *parvulum*

*A parvulum* is frequently isolated from the human oral cavity and found to be associated with oral malodor (halitosis). Research has revealed that patients with IBD, similar to patients with colon cancer, exhibit an accumulation of *A parvulum* in the gut.^9^ Certain oral bacteria (eg, *Atopobium* spp., *Veillonella* spp., *Prevotella* spp., *Streptococcus* spp., and *Aggregatibacter* spp.) are known to liberate hydrogen sulfide (H_2_S), an inflammatory mediator, from sulfur‐containing amino acids.^9^ Investigators identified impaired mitochondrial H_2_S detoxification and the bloom of H_2_S‐producing pathobionts along with the depletion of butyrate‐producing bacteria in the gut of patients with Crohn's disease (CD) by using system biology approaches that combine metagenomic and proteomic data sets.^51^ About one‐quarter of the operational taxonomic units (eg, *Atopobium*, *Fusobacterium*, *Veillonella*, *Prevotella*, *Streptoccocus*, and *Leptotrichia*) that correlate positively with the severity of intestinal disease are known to metabolize sulfur‐containing amino acids into H_2_S. Importantly, *A parvulum* is defined as the key pathobiont, serving the central hub of the H_2_S network. Furthermore, this study demonstrated the colitogenic capacity of *A parvulum* in an *Il10* ^−/−^ colitis model, with the increased expression of the chemokine (C‐X‐C motif) ligand 1 (*Cxcl1*) and *Il17* in the gut, compared with controls, which was mitigated by the administration of the H_2_S scavenger bismuth.^51^ In contrast, *A parvulum* monocolonized germ‐free (GF) *Il10* ^−/−^ mice did not develop significant colitis, suggesting that other microbes, or their metabolites, are required for *A parvulum*–driven colitis. Given the ability of H_2_S to induce proinflammatory molecules (eg, cyclooxygenase (COX)‐2, IL‐8, and CCAAT enhancer binding protein beta [CEBPB])^52^ in epithelial cells and to promote T cell activation,^53^ it is conceivable that *A parvulum* creates niches favorable for the growth of colitogenic pathobionts by inducing H_2_S. At high concentration, H_2_S is a strong inhibitor of cytochrome c oxidase, and hence, mitochondrial oxygen (O_2_) consumption, with deleterious consequences for the epithelial integrity. Furthermore, given that colonocytes obtain more than 70% of their energy from the oxidation of gut bacteria‐derived butyrate,^54^, ^55^ along with the ability of H_2_S to inhibit butyrate oxidation, *A parvulum* may play a role in the epithelial energy deficiency associated with the prevalence of IBD.^56^, ^57^

### 3.4 *Campylobacter* *concisus*

*C concisus* is an oral resident bacteria found in the gut of patients with IBD.^58^, ^59^, ^60^, ^61^ Genomic comparison of oral and enteric *C concisus* strains implies that the enteric strains originate from the oral *C concisus* strains.^62^, ^63^ Although the mechanistic features of the flagellum of *C concisus* are not fully understood, *C concisus* flagellum‐mediated attachment to and invasion of the colonic epithelial cell line Caco‐2 have been documented.^64^ Research has also shown that dense bacterial biofilm formation is common in IBD patients and contributes to the disease pathogenesis through the induction of dysbiosis and resistance to treatment, such as antibiotics.^65^ In this regard, the flagellum of *C concisus* enables it to form biofilm and hence survive in the gut.^66^ In vitro intestinal epithelial cell culture models (eg, Caco‐2, HT‐29/B6 cells) also suggest that *C concisus* can increase intestinal permeability through the dislocation (or downregulation) of ZO‐1, occludin, and claudin‐5, together with apoptotic leaks.^64^, ^67^ Moreover, *C concisus* impairs sodium (Na^+^) absorption in HT‐29/B6 cells through the dysfunction of the epithelial Na^+^ channels.^68^ This is dependent on IL‐32–regulated extracellular signal‑regulated protein kinase (ERK)1/2, as well as claudin‐8–dependent barrier dysfunction, both of which contribute to Na^+^ malabsorption and diarrhea.^68^ *C concisus* also increases the production of proinflammatory molecules such as IL‐8 and COX‐2, which is an enzyme responsible for generating prostaglandins as well as other inflammatory mediators in the intestinal epithelial cells.^69^ In parallel, infected HT‐29 epithelial cells express elevated levels of pattern‐recognition receptors (eg, Toll‐like receptor [TLR] 4, but not TLR2 or TLR5), implicating the role of *C concisus* in modulating the intestinal epithelial responses to bacterial components such as lipopolysaccharide.^69^ In response to *C concisus* colonization of Caco‐2 cells, autophagy‐related genes, such as *ATG9B*, are significantly reduced, implying the importance of escape from autophagy as a bacterial survival strategy within the intracellular compartment.^70^ Interestingly, global gene expression changes in Caco‐2 caused by the exposure to the toxigenic *C concisus* strain AToCC that expresses zonula occludens toxin were distinct from the changes induced by the nontoxigenic strain AICC. The AToCC strain, compared to AICC, induces a more robust expression of genes related to inflammatory responses (eg, IL‐2, IL‐5, IL‐18, CCL2, and TNF signaling) and the pattern recognition receptors involved in sensing intracellular nucleic acids (eg, TLR3), as well as the assembly of the IFI16 inflammasome.^70^

Another *C concisus* virulence factor—membrane‐bound hemolytic phospholipase A2 (PLA2)—exhibits cytolytic effects on Chinese hamster ovary cells in tissue culture, indicating the possible mechanism of cell destruction by *C concisus* during intestinal inflammation.^71^ After passing through the epithelial barrier, *C concisus* can activate immune cells including macrophages and neutrophils in the lamina propria and elicit inflammatory responses. For instance, *C concisus* enhances the production of IL‐8 and TNF‐α by THP‐1 macrophages.^64^ Like the epithelial response against *C conscisus*, genes associated with the host recognition of *C concisus* (eg, those encoding TLRs), as well as inflammasome‐related genes (eg, IFI16, ASC), are significantly upregulated after *C concisus* infection of THP‐1 macrophages.^72^ Also, global gene regulation in macrophages on infection with *C concisus* includes the activation of key inflammatory pathways involving CREB1, NF‐κB, STAT, and interferon regulatory factor signaling.^72^ Further, *C concisus* activates the innate immune system by stimulating CD11b expression in neutrophils, which promotes neutrophil adhesion to the vascular endothelium and an oxidative burst response.^73^ To date, published animal studies with *C concisus* infection are few. The first study, which was conducted in BALB/c mice, showed that the infected mice had marginal gut inflammation with poor colonization.^74^ Another study used antibiotic‐treated IL‐10^−/−^ mice (on the C57BL/6J genetic background) and showed that oral administration of *C concisus* neither induces significant inflammation nor impairs epithelial barrier function in the colon, whereas *C concisus* colonization can cause dysfunction of the epithelial Na^+^ channel associated with watery diarrhea.^68^, ^75^ Despite ample evidence of the colitogenic capacity of *C concisus*, comprehensive animal studies are required to determine the precise impact of gut colonization of *C concisus* on intestinal inflammation.

### 3.5 *Staphylococcus* *aureus*

*S aureus* is a gram‐positive, spherical member of the phylum Firmicutes, and a constituent of the human oral microbiota.^76^, ^77^ Although this bacterium is well characterized by food poisoning through staphylococcal enterotoxin (SE)–mediated mechanisms,^77^, ^78^ patients with CD are also known to have higher levels of *S aureus* in inflamed subgingival sites compared with healthy individuals, even with similar clinical periodontal parameters.^79^ Notably, the increased colonization by this bacterium is also reported in the gut of IBD patients compared with non‐IBD controls.^9^, ^26^ *S aureus* is reported to adhere to intestinal epithelial cells.^80^ It has also been shown that oral administration of *S aureus* strain RN8098, which produces staphylococcal enterotoxin B (SEB), into antibiotic‐pretreated C57BL/6J mice causes epithelial damage in the small, but not the large intestine, whereas no overt inflammation was observed in mice colonized by a SEB mutant strain.^80^ Interestingly, despite the capability of SEs to dampen adherens junction protein expression,^81^ disruption of the adherens junction proteins E‐cadherin and β‐catenin in the small intestine of mice with *S aureus* was detected in both wild‐type and SEB mutant strains. This indicates the possible involvement of virulence factors other than SEB in *S aureus*–induced epithelial damage in the gut.^82^ Furthermore, SEs are known to function as superantigens by binding to the outside of the antigenic peptide binding groove of major histocompatibility complex (MHC)–II on antigen‐presenting cells (eg, macrophages and dendritic cells), as well as to T cell receptors expressing certain Vβ elements.^78^ Thus, the massive proliferation of CD4^+^ T cells with the production of proinflammatory cytokines induced by those interactions may also contribute to the pathogenesis of IBD.

### 3.6 *Klebsiella* spp. and *Enterobacter* spp.

*Enterobacteriaceae* is a large family of gram‐negative bacteria, including *Klebsiella* spp. and *Enterobacter* spp. Most *Enterobacteriaceae* are part of the gut commensal microbiota. However, investigators have shown that colonization of oral‐derived *Klebsiella* spp. (eg, *K pneumoniae*, *K aeromobilis*) isolated from the saliva of patients with CD results in potent Th1 cell differentiation in the gut of gnotobiotic animals.^26^ Importantly, this study showed that oral *Klebsiella* spp. can facilitate the development of Th1‐skewed IBD‐like colitis in IL‐10^−/−^ mice, whereas no overt inflammation was detected in immune‐competent wild‐type B6 mice despite Th1 induction in the gut. Mechanistically, TLR and IL‐18 signaling are required for the *Klebsiella*‐mediated Th1 cell induction through the antigen‐presenting CD11b^+^CD103^+^ dendritic cells. Also, it was shown that upregulation of IFN‐inducible (IFI) genes, such as those encoding guanylate‐binding proteins, CXCL9, MHC‐related molecules, and dual oxidase 2 (Duox2), may facilitate the gut colonization by *K pneumoniae*, as well as the development and recruitment of Th1 cells. Further, the investigators observed that mice lacking IFN receptor 1 failed to respond to the *K pneumoniae* colonization. These results imply that Th1 responses triggered by *K pneumoniae* are sustained via an IFI‐mediated feed‐forward loop.^26^ Of note, these oral‐derived *Klebsiella* isolates are resistant to multiple antibiotics, indicating the potential risk of antibiotic use in a clinical setting, as such a regimen may allow the bacteria to colonize the gut and induce colitis in IBD‐susceptible hosts.

Ample evidence of the clinical association between periodontitis and IBD^13^ prompted us to assess the impact of periodontitis on intestinal inflammation. Our recent study revealed the deleterious contribution of periodontitis, associated with the expansion of oral pathobionts belonging to the *Enterobacteriaceae* family in the oral cavity, to the development of distant intestinal inflammation.^83^ In this study, by combining ligature‐induced murine periodontitis and DSS‐induced colitis models, we revealed that oral inflammation fosters blooms of *Enterobacteriaceae* including *Klebsiella* spp. and *Enterobacter* spp. and enforces colonization of these oral pathobionts in the gut of genetically susceptible IL10^−/−^ mice (but not wild‐type B6 mice), resulting in exacerbation of intestinal inflammation. Further, we showed that direct gut colonization of these oral pathobionts strongly induces colonic IL‐1β production by activating the inflammasome pathway in intestinal macrophages in the inflamed gut, thereby aggravating the intestinal pathology.^83^ Importantly, an overt increase of oral pathobionts did not occur in the healthy gut, even in the mice with periodontitis, implying that at least two hits (ie, prerequisites) to the microbiotas in the mouth and gut are essential for the development of oral pathobiont‐driven intestinal inflammation. The first prerequisite is oral dysbiosis, which drastically increases the number of oral pathobionts in the oral cavity, and thus increases the chance of successful transmission to the intestine. As discussed, the physiological barrier functions of the gastrointestinal tract, particularly in the stomach, deter the successful transmission of ingested bacteria. Given the bactericidal effect of gastric acids, the expansion of oral pathobionts in the dysbiotic oral microbiota must be achieved to increase the chance of bacterial survival in the stomach, followed by the successful translocation to the intestine. Attenuation of gastric acidity in patients with IBD or the inhibition of acid secretion may explain why amassed oral bacteria are often found in the gut of IBD patients.^10^, ^11^, ^84^, ^85^ The second prerequisite involves the disruption of gut colonization resistance conferred by gut dysbiosis, which may be required to enable oral pathobionts (that successfully passed through the gastric barrier) to colonize the gut. In our study, gut inflammation dampened colonization resistance provided by the gut commensals and allowed ingested oral pathobionts (eg, *Klebsiella* spp.) to successfully colonize the gut.^83^ Given that the inflammatory milieu favors the growth of members of the *Enterobacteriaceae* family,^86^, ^87^, ^88^ intestinal inflammation may also be a potent driving factor that instigates the ectopic colonization of certain types of oral pathobionts such as *Klebsiella* spp. that can gain growth benefits in the inflamed gut. Consistent with the previous report of IBD patient‐derived oral *Klebsiella* spp.,^26^ the *Klebsiella* strains that we isolated (eg, *K aerogenes*) from periodontitis mice also have antibiotic resistance (data not shown), indicating the potential risk of antibiotic use in the development of gut inflammation, which is mediated by ectopically colonized oral pathobionts in the dysbiotic gut environment.

### 3.7 Other oral bacteria

Like *Atopobium* spp., certain oral bacteria (eg, *Veillonella* spp.) enriched in the gut of IBD patients have been identified as major producers of H_2_S, implicating their proinflammatory potential in the gut.^10^, ^89^ Also, other indigenous oral bacteria (eg, *Streptococcus* spp. and *Neisseria* spp.) can produce acetaldehyde by catabolizing ethanol and glucose.^90^ Given the proinflammatory capacity of acetaldehyde through disruption of the epithelial barrier function,^91^, ^92^, ^93^ it is possible that ectopic colonization of the gut by these oral bacteria could instigate gut inflammation. Furthermore, besides the enteral colonization described in Figure 1, certain types of oral pathobionts, such as *Streptococcus mutans*, may impact the intestinal pathology through hematogenous spread from the oral cavity. *S mutans* has virulence factors associated with the etiology and pathogenesis of dental caries.^94^, ^95^ Also, a higher prevalence of dental caries and higher salivary counts of *S mutans* are reported in CD patients compared to the control group.^96^ Several *S mutans* strains isolated from the oral cavity of patients with ulcerative colitis (UC) caused aggravation of murine DSS‐induced colitis, suggesting the potential involvement of highly virulent *S mutans* in the occurrence of UC.^97^ In this study, the investigators found that intravenous administration of TW295, a serotype *k* strain of *S mutans* expressing collagen‐binding protein, can specifically colonize the liver, rather than the intestine, and induce IFN‐γ production (presumably from the hepatocytes), thereby increasing the susceptibility to DSS‐colitis. As oral administration of TW295 did not produce colitis aggravation, it is conceivable that certain oral pathobionts, such as *S mutans*, coming from the circulating blood, but not from the mucosa surrounding the lumen of the gastrointestinal tract, are involved in the aggravation of colitis. Consistently, it is reported that *S mutans* can disseminate to the systemic circulation in individuals who have had dental procedures (eg, orthodontics, tooth extraction) or oral disease (eg, oral cancer).^9^

## 4 IMMUNOLOGICAL PATHWAY (VIA TRANSLOCATION OF ORALLY PRIMED IMMUNE CELLS TO THE GUT)

**FIGURE 2:**
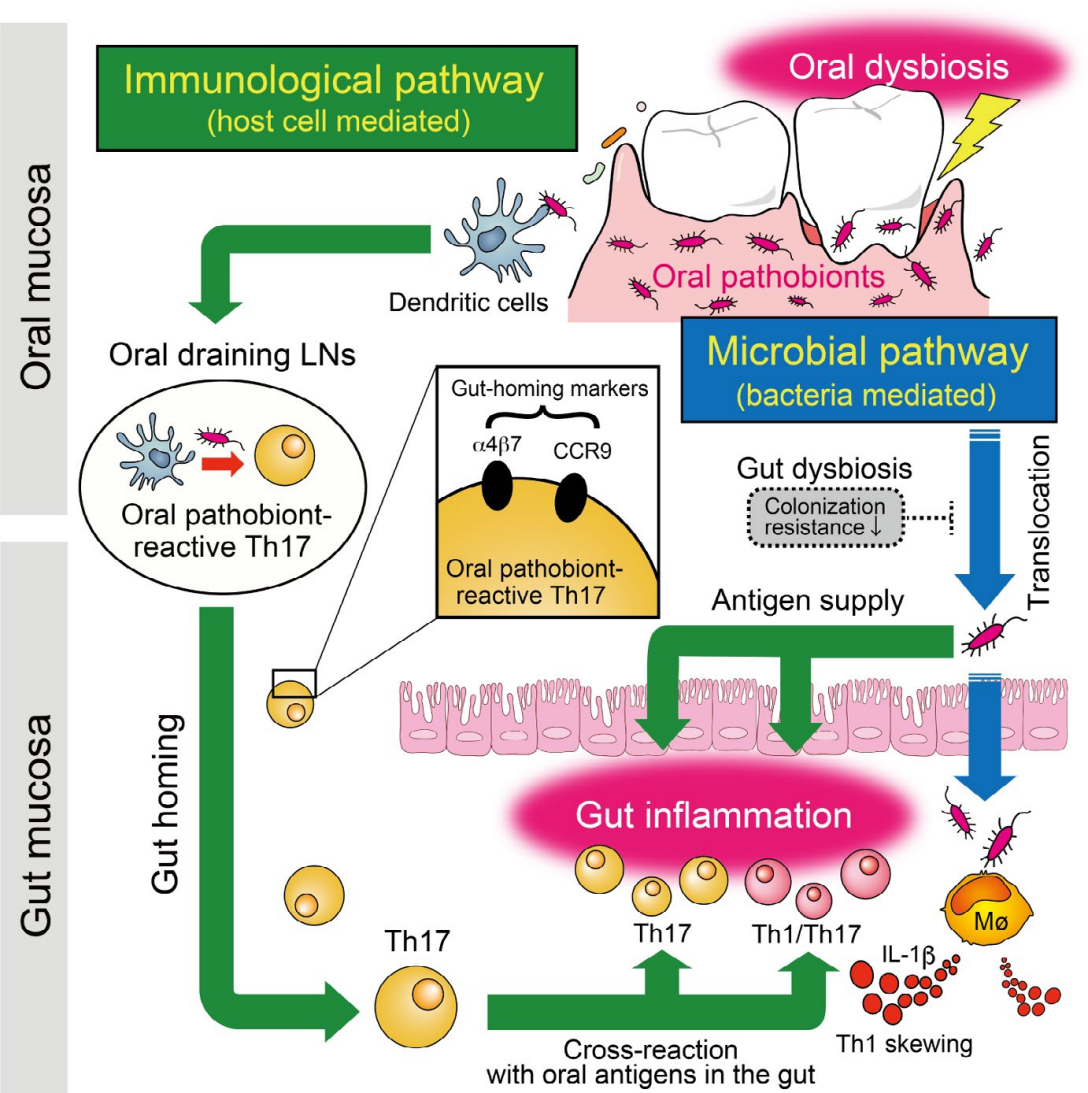
Possible mechanisms of gut inflammation mediated by transmigration of orally primed T cells to the gut (immunological pathway). In parallel with the direct translocation of oral pathobionts to the gut provoked by concurrent oral and gut dysbiosis (Figure 1, microbial pathway), the transmigration of oral immune cells to the gut also plays a key role in the mouth–gut axis during the pathogenesis of oral pathobiont‐driven colitis (ie, the immunological pathway). Mechanistically, during periodontal inflammation, orally primed Th17 cells that recognize oral pathobionts (eg, *K aerogenes*) are generated in the oral draining lymph nodes (LNs). Oral pathobiont‐reactive Th17 cells express gut‐homing molecules such as CCR9 and α4β7. When Th17 cells of oral origin reach the gut, they can be activated by translocated oral pathobionts and promote the development of colitis. Given the phenotypic changes of oral Th17 cells toward Th1, such as Th17 cells in the gut of mice with periodontitis and the concurrent presence of Th1 skewing factor IL‐1β (produced by intestinal macrophages exposed to oral *K aerogenes*, as evident in the microbial pathway, Figure 1), it is likely that the microbial and immunological pathways synergistically aggravate the intestinal pathology during the oral pathobiont‐driven gut inflammation

Ample evidence demonstrates that immune cells can move from the gut to other organs (eg, liver, kidney, joints) and contribute to the disease pathogenesis at distant sites.^98^, ^99^, ^100^ The immune cell trafficking between the gut and other organs seems to be bidirectional. It is reported that leukocytes in the oral draining lymph nodes, particularly the cervical lymph nodes (cLNs), can travel to the gut even under steady‐state conditions,^101^ indicating the potential role of systemic immune cell circulation in human health and disease. In this context, we unveiled the mechanistic link between the mouth and gut during the development of gut inflammation from an immunological point of view^83^ (Figure 2).

As mentioned above, ligature‐induced murine periodontitis increases the susceptibility to acute DSS‐induced colitis through the direct gut colonization by oral pathobionts^83^ (Figure 1). Interestingly, even though the acute DSS‐induced colitis model may lack sufficient time to develop T cell immunity in the gut, we observed a prominent increase of Th17 and Th1 cells in the colonic mucosa of ligature–DSS mice compared with DSS colitis only mice. Given the known cellular trafficking between the oral cavity and the gastrointestinal tract^101^ and the role of Th17 in periodontal inflammation,^102^ we hypothesized that the pathogenic T cells that accumulate in the gut of ligature–DSS mice originate from the oral cavity. To this end, we first characterized the immune responses provoked by periodontitis in the oral cavity. Then, we showed that CD3^+^CD4^+^CD44^hi^CD62L^lo^ effector memory T (T_EM_) cells are enriched in the cLNs of mice that developed periodontitis. In accordance with a previous report,^103^ we observed that T_EM_ cells accumulated in periodontitis mice display the IL‐17A–producing RORγt^+^ Th17 phenotype. By coculturing oral antigen‐pulsed dendritic cells (DCs) and isolated orally primed Th17 cells, we discovered that oral Th17 T_EM_ cells were reactive to oral pathobionts, including *Klebsiella* spp. and *Enterobacter* spp., all of which expanded in the inflamed, but not the healthy, oral mucosa. These results suggested that oral pathobiont‐reactive Th17 cells are generated during periodontitis, raising the question of whether oral Th17 cells can travel to the gut. Further analysis showed the cell surface expression of gut‐homing markers α4β7 integrin and CCR9 on these oral Th17 cells, indicating their gut tropism. To obtain direct evidence of the transmigration of oral Th17 cells to the gut, we used in vivo photoconversion of cells in the cLNs of transgenic mice expressing the Kaede protein^104^ and monitored the ability of these cells to migrate to the gut. In this trafficking system, all cells in Kaede mice constitutively express the photoconvertible Kaede green fluorescent protein. When the photoconvertible protein is exposed to violet light, the cell color changes from Kaede green to Kaede red.^104^ As previously reported,^101^ we detected Kaede red CD4^+^ T cells in cLNs in the steady‐state gut, providing concrete evidence of the transmigration of orally primed Th17 cells to the gut mucosa. Interestingly, the influx of oral Th17 cells to the gut was significantly increased in mice with DSS‐induced colitis. Although the precise mechanisms of this transmigration remain unclear, the upregulation of mucosal addressin cell adhesion molecule 1 (MadCAM‐1), a ligand for α4β7 integrin expressed in vessels in the colonic lamina propria of patients with IBD and experimental animal models including DSS‐induced colitis models,^105^, ^106^, ^107^ suggest that an enhanced interaction between α4β7 integrin and MadCAM‐1 plays a role in accelerating the influx of oral Th17 cells into the inflamed gut (Figure 2). To validate the colitogenic capacity of oral Th17 cells in the gut, we conducted multiple immune cell experiments, including adoptive transfer colitis. We found that isolated oral Th17 cells (ie, Kaede red cells isolated from the gut of ligatured mice) induced colitis when transferred intravenously into *Rag1* ^−/−^ mice colonized by the oral pathobiont *K aerogenes* in the gut associated with an increase of Th17 cells (RORγt^+^) and Th1/Th17 cells (RORγt^+^ T‐bet^+^); in contrast, Kaede green cells isolated from the gut of ligatured mice failed to cause colitis. Interestingly, administration of IL‐1 receptor antagonist (anakinra) ameliorated the severity of colitis in the Kaede red cell–transferred mice. Considering the known role of IL‐1β in skewing Th17 cells toward Th1 phenotypes, intestinal IL‐1β induced by the gut colonization of oral pathobionts (eg, *K aerogenes*, Figure 1) not only induces proinflammatory innate lymphoid cells and Th17 cells,^108^ but also acts as a Th1 skewing factor for generating Th1/Th17 cells, which also accumulate in the gut of individuals with IBD^109^, ^110^, ^111^, ^112^, ^113^ (Figure 2). In our study, in accordance with the current understanding of a key role of Th17 cells in the commensal‐driven oral inflammation,^103^, ^114^, ^115^, ^116^, ^117^ we observed the prominent increase of oral pathobiont‐reactive Th17 cells in the oral cavity in response to the ligature‐induced periodontitis.^83^ In this context, despite the evidence that commensal‐reactive Th17 cells generated in the gut are not pathogenic,^118^ IFN‐γ‐secreting Th1‐like exTh17 cells that arise from Th17 cells under certain circumstances in the gut can induce severe intestinal inflammation.^109^, ^119^ Interestingly, while the oral commensal pathobiont‐reactive Th17 cells that arise during periodontitis exhibit a Th17 phenotype (RORγt^+^ T‐bet^−^) associated with IL‐17A but not IFN‐γ production, when these oral Th17 cells reach the gut mucosal compartment they seem to acquire a Th1‐like Th17 phenotype (RORγt^+^ T‐bet^+^) associated with IFN‐γ production (Figure 2).^83^ Considering the clinical importance of Th1/Th17 cells, this functional conversion of orally generated Th17 cells into pathogenic Th1/Th17 cells in the gut microenvironment may be an important area for future research.

## 5 PERSPECTIVES

**FIGURE 3:**
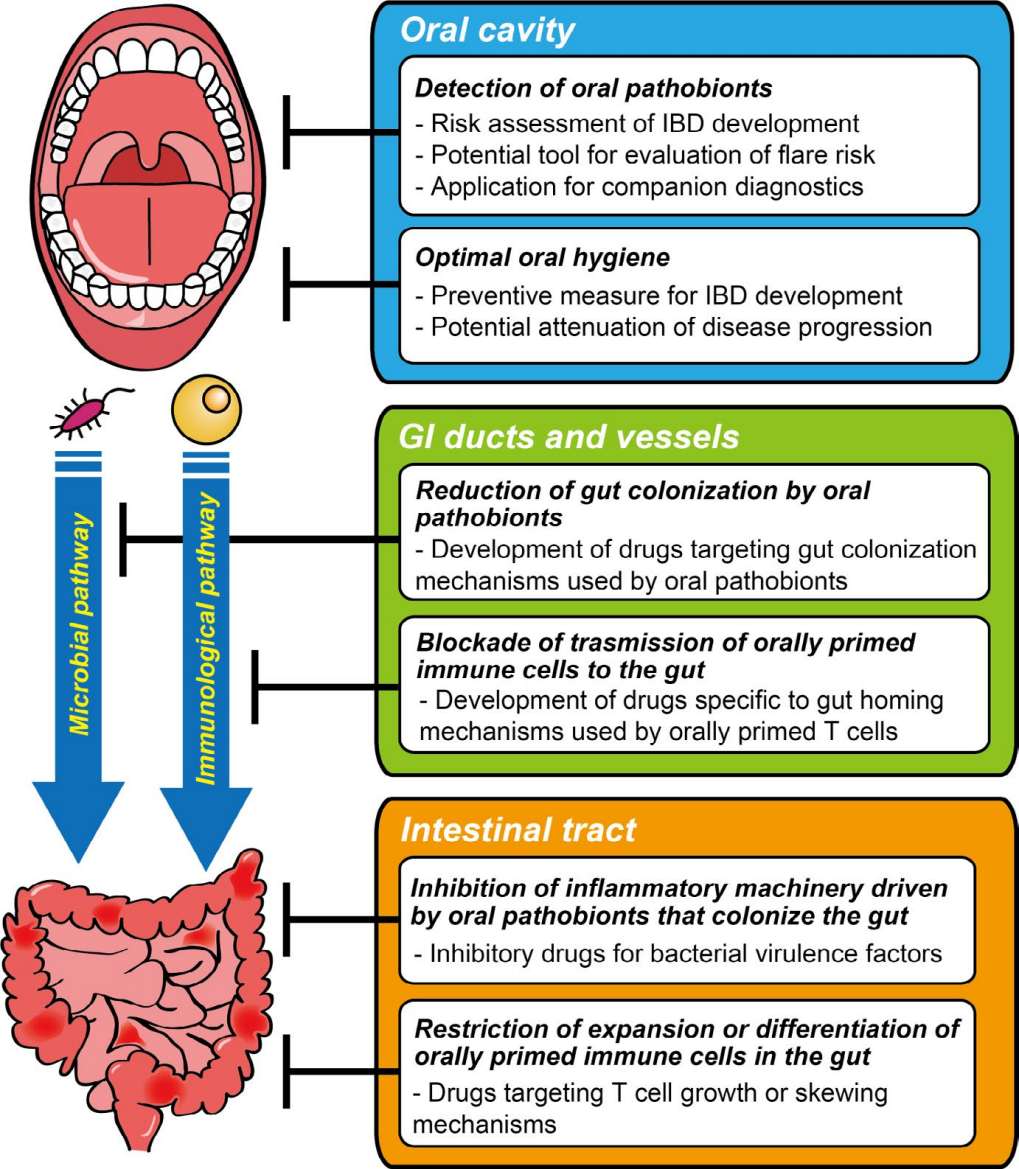
Potential approaches to the development of IBD interventions by targeting the oral–gut axis. The oral–gut axis can be divided into at least three targetable interfaces: (1) the oral cavity where oral pathobionts and potentially pathogenic immune cells are generated, (2) the gastrointestinal (GI) ducts and vessels that are used for the trafficking of oral–derived pathogenic agents to the gut, and (3) the intestinal tract where oral‐derived pathogenic agents can be virulent. Each interface holds potential for the development of clinical interventions in the treatment of IBD

Over the past decade, the research field of oral microorganisms and intestinal inflammation has been dramatically expanded by studies that primarily focus on the impact of direct colonization of oral pathobionts in the gut (Figure 1, microbial pathway). Furthermore, the use of murine models has revealed the novel aspects of the complex intermucosal connection between the mouth and the gut. Orally primed pathogenic T cells can transmigrate to the gut, where they are reactivated by ingested oral pathobionts, and thus, exacerbate intestinal inflammation (Figure 2, immunological pathway). Yet, despite advances, major knowledge gaps still exist. For example, the considerable microbial dissimilarity between humans and mice^120^ challenges the extent to which our findings in the realm of murine studies are readily translatable to humans. In this regard, the colitogenic murine oral pathobionts (eg, *K aerogenes*) that we identified are genetically very similar to *K aeromobilis*, which is a strong Th1‐inducing colitogenic oral pathobiont isolated from the saliva of IBD patients.^83^, ^121^ Although the detailed mechanisms remain unexplored, the genetic similarity of these species, and their functional similarity, considering the induction of Th1‐biased immunity during gut inflammation, suggest that the immunological interaction mediated by oral pathobiont‐reactive immune cells contributes to the pathogenesis of intestinal inflammation in human IBD. At present, neither the class of drugs, nor specific drugs, that target the oral–gut axis are available to treat patients with intestinal inflammation. Future investigations of the oral cavity will lead to a better understanding of the essential steps in the development of novel biomarkers and therapeutics for intestinal inflammation (Figure 3, Oral cavity). For instance, early detection of certain oral pathobionts may help to identify individuals at high risk of the development or relapse of IBD. Also, optimal oral hygiene to reduce the supply of oral pathobionts may attenuate ongoing disease progression in the gut, as well as prevent the development of IBD. A focus on the mode of transmission of oral pathobionts and oral immune cells to the gut could inspire the development of another potential intervention (Figure 3, GI ducts and vessels). For the microbial pathway, this could be achieved by reducing the chance of gut colonization by oral pathobionts through the proper use of PPIs or antibiotics to preserve the physiological barrier functions in the stomach and gut against the invasion of extraintestinal bacteria. In fact, PPI exposure has been associated with adverse clinical consequences (eg, IBD‐related hospitalization or surgery) in patients with both UC and CD.^122^, ^123^ Further, IBD patients treated with PPIs have been reported to be less likely to achieve remission while taking infliximab.^124^ For the immunological pathway, intervention could be achieved by blocking the transmission of orally primed immune cells (eg, pathogenic oral Th17 cells) by inhibitors or biologics specific to the molecules that guide the oral‐derived immune cells to gut. In this context, anti‐α4β7 integrin therapy has been shown to be effective in moderate‐to‐severe CD.^125^, ^126^ Given the expression of α4β7 on orally primed T cells, the improvement of disease outcomes may be due, in part, to the inhibition of the transmigration of pathogenic orally primed T cells to the gut. Consequently, there remains an unmet need to reliably predict the efficacy of anti‐integrin therapy to maximize the cost‐effectiveness by determining responders and nonresponders. Thus, a better understanding of the immunological link between the mouth and gut of IBD patients may influence clinical decision‐making regarding treatment choices. Furthermore, it would be useful to elucidate the precise mechanisms by which oral‐derived pathobionts and immune cells exacerbate gut inflammation. This may pave the way to develop novel clinical options for IBD (Figure 3, Intestinal tract). Clearly, further research of the complex inflammatory machinery driven by oral pathobionts in the gut (eg, identification of virulence genes, regulatory mechanisms, and downstream immune activations) will become a basis for the future development of novel therapy for IBD.

The microbial and immunological connection between the mouth and the gut in the development of intestinal inflammation continues to be an area of intense study. From the clinical standpoint, larger cohorts and longitudinal studies are required to evaluate the importance of the oral–gut axis during the development of intestinal inflammation. In parallel, from the perspectives of basic and translational science, further characterization of the microbial and immune profiles of both sites and the factors affecting the gut colonization by oral pathobionts may present opportunities to develop unique and effective therapies for IBD.

## CONFLICT OF INTEREST

The authors declare no competing interests.
